# Laryngoscopic findings secondary to Influenza A infection: Report of two cases

**DOI:** 10.1016/j.bjorl.2026.101880

**Published:** 2026-07-22

**Authors:** Gustavo Polacow Korn, Renata Rangel Azevedo, Octavio Sakahara Saito, Isadora Serotini Pertinhez

**Affiliations:** Universidade Federal de São Paulo, São Paulo, SP, Brazil

## Introduction

Influenza A infection may cause intense coughing for a few days, usually up to one week.^1^ Intense coughing is a known cause of phonotrauma, and may lead to severe dysphonia, including aphonia. Cases of aphonia caused by coughing in the presence of Influenza A are not common in literature.

We present two cases of Influenza A patients who experienced severe coughing leading to phonotrauma, aiming to highlight early recognition and management of this atypical presentation with distinct laryngeal findings.

## Case study reports

**Fig. 1:**
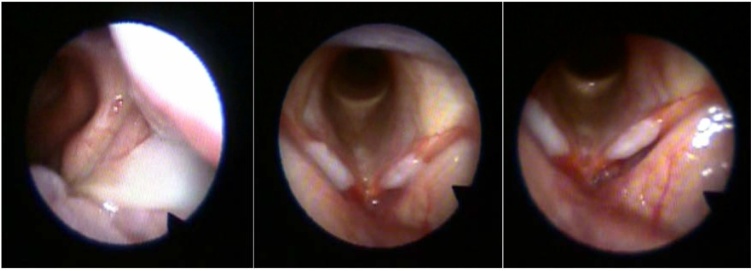
Nasofibrolaryngoscopy of Case 1 at the first assessment. The image on the left shows secretion in the left middle meatus; the central image shows the initial aspect of the larynx (vocal folds with preserved mobility, marked hyperemia, and a whitish, opaque, elevated area involving the middle third of the membranous portion and extending to part of the anterior and posterior thirds of both vocal folds); the image on the right was obtained with the device positioned closer to the glottis, where the elevated lesion can be observed.

**Fig. 2:**
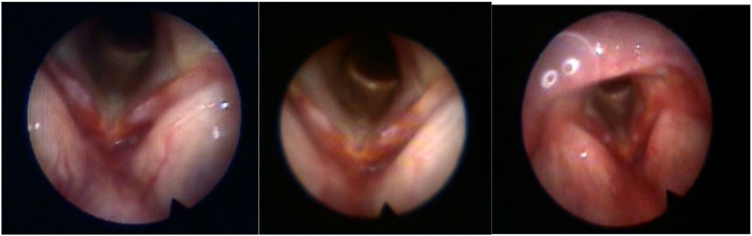
Nasofibrolaryngoscopy of Case 1. The left image taken one week after treatment; the central image taken after two weeks; and the image on the right taken one month later.

Case 1 67-year-old male patient, an anesthesiologist, who reported discrete coughing and shortness of breath for a day. He went to the Emergency Care Unit and was admitted to hospital for six days after being diagnosed with Influenza A and treated with Oseltamivir. He also received Azithromycin and Ceftriaxone. As of the third day in hospital, he became aphonic due to increased coughing. He was assessed ten days after the onset of symptoms. Nasofibrolaryngoscopy revealed a yellowish secretion arising from the left middle meatus in the direction of the choanae, and in the larynx, vocal folds with preserved mobility and important hyperemia and a whitish opaque area, elevated, in the middle third of the membranous portion and part of the anterior and posterior thirds of both vocal folds (Fig. 1). Due to the diagnosis of associated rhinosinusitis, he was treated with Clavulanate, oral and nasal steroids and a Pantoprazole. Also, absolute voice rest for five days was recommended, and relative rest for the following two days, with reassessment in a week. He was reassessed a week later, with an important improvement in voice and aspect of the laryngoscopy (Fig. 2). The patient was then referred to speech therapy.

**Fig. 3:**
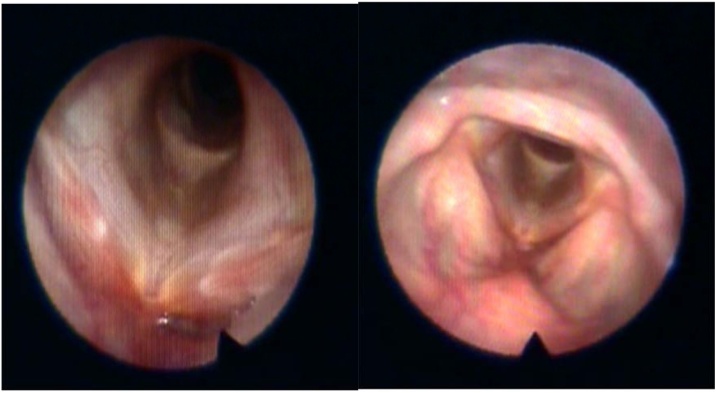
Nasofibrolaryngoscopy of Case 1, image on the left taken 45-days after treatment, and image on the right taken after three months, with resolution of lesions.

Vocal evaluation revealed the presence of dysphonia (G2R2B1A0S2I1), and important pneumo-phono-articulatory incoordination, laryngopharyngeal resonance and great phonatory tension. During 12 speech therapy sessions, aspects related to voice care (vocal hygiene) and strategies to deal with coughing were dealt with. Within three months the patient improved completely from the dysphonia and the cough, without any lesion in the larynx (Fig. 3).

**Fig. 4:**
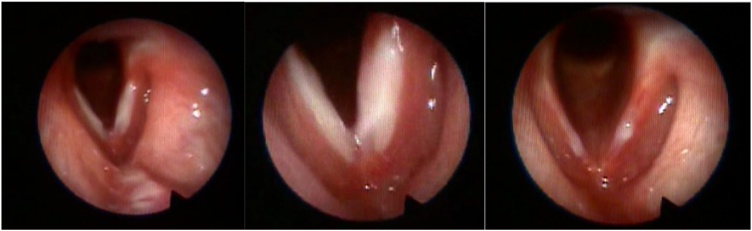
Nasofibrolaryngoscopy of Case 2. On the left, image of the larynx at the first assessment, showing vocal folds with marked hyperemia and a whitish, opaque, elevated area involving the entire membranous portion of both vocal folds; central image obtained with the device positioned closer to the glottis; image on the right obtained one week after treatment.

Case 2 16-year-old male patient, a student, referring cough, fever and headache for ten days when he was in the United States, where he tested positive for Influenza A one day after beginning of the symptoms and was treated with Oseltamivir. His symptoms improved, except for the cough for five days, and he reported becoming aphonic after coughing heavily. In the office, nasofibrolaryngoscopy combined with telelaryngoscopy revealed a yellowish secretion arising from the right middle meatus in the direction of the choanae, and in the larynx, vocal folds with preserved mobility, with important hyperemia and a whitish opaque area, elevated, in all the membranous portion of both vocal folds. The presence of a secretion in the lingual surface of the epiglottis probably arising from nasal cavities is also noted. He was treated with Cefuroxime Axetil, oral and nasal steroids and a gastric protector. Also, absolute voice rest for five days was recommended, and relative rest for two days, with reassessment in a week. A week later he was reassessed, with an important improvement in voice and aspect of the laryngoscopy. He was asked to return in ten days, but did not follow up as suggested. His mother was contacted by telephone three months later and reported that his coughing was over, and his voice was back to normal (Fig. 4).

## Discussion

Cases of upper respiratory tract infection due to Influenza A are usually seen in the emergency room without the assessment of an otolaryngologist. We highlight that in cases where coughing results in vocal quality worsening it is fundamental to undergo otorhinolaryngologic assessment. Based in our cases, we strongly suggest a early laryngoscopic evaluation because a vocal fold lesions may develop. In both cases, hyperemia and a whitish elevated opaque area were found in the membranous portion of both vocal folds. Early speech treatment promotes healing and guards against long-term dysphonia. Postponing laryngoscopic examination may cause dysphonia to worsen over time.

Both patients were without dysphonia after three months. It seems that no long-term negative vocal effects were observed.

We have not found reports of lesions in the membranous portion of the vocal folds in patients with acute coughing in literature. Regarding chronic cough, Shaha et al.^2^ have reported five cases of lesions of vocal fold coverage and Adessa et al.^3^ in a retrospective study of 419 patients found lesions in the membranous portion in 3% of the cases.

Besides voice therapy focused on the improvement of coverage alteration of vocal folds, it is important to focus on care to manage coughing, thus offering a direction for a fast recovery.

Nasofibrolaryngoscopy also enables assessment of other causes of coughing, such as acute rhinosinusitis, present in both cases. It is important to point out that even when treating a viral infection, the possibility of a bacterial co-infection should be considered. The presence of acute rhinosinusitis with subsequent postnasal drip in both patients may also have contributed to the severity of coughing, which in turn led to the development of vocal fold lesions.

Vocal fold lesions in these cases may be attributed to viral involvement, rhinosinusitis with subsequent postnasal drip, and severe coughing. More research is required to determine how severe viral cough as Influenza affects the larynx. It is essential that these cases be systematically documented and reported.

## Conclusion

We present two patients diagnosed with Influenza and significant dysphonia. We believe that vocal fold lesions in these cases may be attributed to viral involvement, rhinosinusitis, with subsequent postnasal drip, and severe coughing resulting in phonotrauma.

## ORCID IDs

Gustavo Polacow Korn: 0000-0003-3718-204X

Renata Rangel Azevedo: 0000-0003-3757-5687

Octavio Sakahara Saito: 0000-0002-2443-6189

Isadora Serotini Pertinhez: 0009-0005-1649-6287

## Data availability statement

The authors declare that all data are available in repository.

## Declaration of competing interest

The authors declare no conflicts of interest.
